# Interpretable weakly-supervised learning through kernel density matrices: A digital pathology use case

**DOI:** 10.1371/journal.pone.0335826

**Published:** 2025-11-05

**Authors:** Sebastian Medina, Eduardo Romero, Angel Cruz-Roa, Fabio A. González

**Affiliations:** 1 MindLab Research Group, Universidad Nacional de Colombia, Bogotá, Colombia; 2 The Wallace H. Coulter Department of Biomedical Engineering at Georgia Tech and Emory University, Atlanta, Georgia, United States of America; 3 Computer Imaging and Medical Applications Laboratory (CIM@LAB), Universidad Nacional de Colombia, Bogotá, Colombia; 4 GITECX & AdaLab research groups, Universidad de los Llanos, Villavicencio, Colombia; Instituto Butantan, BRAZIL

## Abstract

Classification methods based on deep learning require selecting between fully-supervised or weakly-supervised approaches, each presenting limitations in uncertainty quantification and interpretability. A framework unifying both supervision modes while maintaining quantifiable interpretation metrics remains unexplored. We introduce WiSDoM (Weakly-Supervised Density Matrices), which uses kernel matrices to model probability distributions of input data and their labels. The framework integrates: (1) differentiable kernel density matrices enabling stochastic gradient descent optimization, (2) local-global attention mechanisms for multi-scale feature weighting, (3) data-driven prototype generation through kernel space sampling, and (4) ordinal regression through density matrix operations. WiSDoM was validated through supervised patch classification (κ = 0.896) and weakly-supervised whole-slide classification (κ = 0.930) on histopathology images. WiSDoM generates three quantifiable outputs: posterior probability distributions, variance-based uncertainty maps, and phenotype prototypes. Through validation in a Gleason grading task at a patch and whole-slide level using histopathology images, WiSDoM demonstrated consistent performance across supervision modes (κ > 0.89) and prototype interpretability (0.88 expert agreement). These results show that kernel density matrices can serve as a foundation for classification models requiring both prediction interpretability and uncertainty quantification across supervision modes.

## Introduction

Deep learning techniques have demonstrated effectiveness in various classification tasks, yet they demand choosing between fully-supervised approaches requiring detailed annotations and weakly-supervised methods using limited labels. The primary approach to weakly supervised learning is Multiple Instance Learning (MIL), where input data is divided into small segments that inherit global labels. However, this method often leads to a loss of context and information. Moreover, these models are commonly seen as “black boxes" due to their complex decision-making processes [1].

Attention-guided weakly-supervised MIL methods have attempted to address these limitations. By incorporating attention mechanisms, these methods improve data aggregation and model interpretability through attention heatmaps [2,3] and region highlighting [4]. Despite these improvements, full model transparency and unified supervision approaches remain open challenges.

This research introduces WiSDoM (Weakly Supervised Interpretable Density Matrices), a probabilistic framework that combines kernel density matrices (KDM) [5] with attention mechanisms. WiSDoM models probability distributions through kernel methods integrated with deep neural networks, enabling both fully-supervised and weakly-supervised learning within the same mathematical framework. The KDM approach has previously shown effectiveness in supervised medical image classification tasks [6].

Computational pathology presents an ideal testing ground for this framework, as it requires both interpretability and flexible supervision approaches [7]. Current applications range from cellular analysis [8] and tissue segmentation [9] to outcome prediction [10–12] and biomarker discovery [13–16]. In this field, weakly supervised methods have enabled slide-level tasks including cancer grading [17,18], tumor subtyping [4], and metastasis detection [19].

Prostate cancer (PCa) classification particularly exemplifies these challenges. As the second most common cancer in men (1.2 million cases, 350,000 deaths annually) [20], its diagnosis depends on microscopic tissue analysis through the Gleason grading system [21]. While the International Society of Urological Pathology (ISUP) standardized this into five groups [22], significant observer variability persists [23], affecting treatment decisions [24–26].

We hypothesize that WiSDoM’s unified supervision approach and interpretable outputs can address both the annotation burden and transparency limitations in computational pathology, using prostate cancer grading as a validation case. This leads to the following contributions:

- An extension of KDM to a weakly-supervised framework, leveraging interpretability in these scenarios
- Enhancement of interpretability with heatmaps which highlight diagnostically relevant regions while producing phenotypic prototypes from its latent space
- A comprehensive understanding of the model’s decision-making process at estimating uncertainty
- Validation including both fully-supervised and weakly-supervised settings, showing WiSDoM’s ability to adapt to different levels of supervision.

## Materials and methods

### Density matrices

Density matrices are a mathematical tool to describe the state of a quantum system and model quantum uncertainty. However, uncertainty can be classical as well, in that case, the quantum system can be referred to as a statistical mixture of different states denoted as $|\psi_{i}\rangle$, each associated with a probability *p*_*i*_. The probabilities *p*_*i*_ satisfy the condition $\sum_{i}^{N}p_{i}=1$. To represent this statistical mixture, we define a density matrix, *ρ*:

$$\rho =\sum_{i}^{N}p_{i}\left|\psi_{i}\right\rangle \left\langle \psi_{i}\right|$$ (1)

where $\left\langle \psi_{i}\right|$ represents the conjugate transpose of $|\psi_{i}\rangle$. Computing the probability of finding a system with the state represented by *ρ* in a state |ψ⟩ after a measurement can be defined as:

$$p(|\psi \rangle |\rho )=\mathrm{Tr}(|\psi \rangle \langle \psi |\rho )=\langle \psi |\rho |\psi \rangle =\sum_{i}^{N}p_{i}{\left|\left\langle \psi |\psi_{i}\right\rangle \right|}^{2}$$ (2)

Density matrices are effective tools for expressing probability distributions and carrying out various computations with efficiency. They enable the determination of outcomes for quantum measurements, expected values, among others.

### Kernel density matrix

A KDM, can be sought as a density matrix defined in the Hilbert space induced by a kernel. KDMs can be used to efficiently represent joint probability distributions and to perform inference, generation and sampling. Since the definition of all the operations inside KDMs are differentiable, they can be integrated into deep learning models. The formal definition of KDM is as follows, as originally stated by the authors [27]:

**Definition 1** (Kernel Density Matrix). *A Kernel Density Matrix over a set* 𝕏 *is a triplet* $\rho =\left(C,p,k_{\theta }\right)$ *where* $C=\left\{x^{\left(1\right)},\ldots ,x^{\left(m\right)}\right\}\subseteq 𝕏,p=\left(p_{1},\ldots ,p_{m}\right)\in {\mathbb{R}}^{m}$ *and* $k_{\theta }:𝕏\times 𝕏\to \mathbb{R}$, *such that* $\forall x\in 𝕏,k(x,x)=1,\forall ip_{i}\geq 0$ *and* $\sum_{i=1}^{n}p_{i}=1$

The elements of *C* are the components of the KDM, and the *p*_*i*_ value represents the mixture weight, or probability, of the component $x_{i}$. If $\phi :{\mathbb{R}}^{n}\to ℋ$ is the mapping to the reproducing kernel Hilbert space (RKHS) ℋ associated to the kernel $k_{\theta },\rho$ represents a density matrix defined as in Eq (1) with components $\left|\psi_{i}\right\rangle =\left|\phi \left(x^{\left(i\right)}\right)\right\rangle$. The projection function associated to a KDM *ρ* is defined as:

$$f_{\rho }(x)=\sum_{x^{\left(i\right)}\in C}p_{i}k_{\theta }^{2}\left(x,x^{\left(i\right)}\right)$$ (3)

The projection function in Eq (3) can be transformed in a probability density function (PDF) by multiplying it by a normalization constant that depends on the kernel of the KDM:

$${\hat{f}}_{\rho }(x)={ℳ}_{k}f_{\rho }(x)$$ (4)

### Inference with Kernel density matrices

Inference involves estimating unknown output variables based on known input variables and a model’s parameters. A probabilistic approach characterizes the input-output relationship as a probability distribution, such as $p\left({𝐱}^{\prime },{𝐲}^{\prime }\right)$, which captures the uncertainty inherent in the data generation process. When predicting output variables, both sources of uncertainty must be taken into account and incorporated into the output distribution, p(𝐲). KDM inference transforms the probability distribution of input variables, p(𝐱), into a distribution of output variables, p(𝐲), by utilizing a joint probability of inputs and outputs, $p\left({𝐱}^{\prime },{𝐲}^{\prime }\right)$. With KDM we can represent these probability distributions as follows:

$$\rho_{𝐱}=\left({\left\{x^{\left(i\right)}\right\}}_{i=1\ldots m},{\left(p_{i}\right)}_{i=1\ldots m},k_{𝕏}\right)$$ (5)

$$\rho_{{𝐱}^{\prime },{𝐲}^{\prime }}=\left({\left\{\left(x^{\prime (i)},y^{\prime (i)}\right)\right\}}_{i=1\ldots m^{\prime }},{\left(p_{i}^{\prime }\right)}_{i=1\ldots m^{\prime }},k_{𝕏}⊗k_{𝕐}\right)$$ (6)

$$\rho_{𝐲}=\left({\left\{y^{\prime (i)}\right\}}_{i=1\ldots m^{\prime }},{\left(p_{i}^{\prime \prime }\right)}_{i=1\ldots m^{\prime }},k_{𝕐}\right)$$ (7)

The parameters of the inference model correspond to the parameters of the KDM $\rho_{{𝐱}^{\prime },{𝐲}^{\prime }}$. These parameters can be estimated in a non-parametric way which does not scale well to large datasets, discriminative learning by performing gradient-based optimization minimizing a suitable loss function like cross-entropy loss or mean square error depending on the output variable type and task, and maximum likelihood learning which estimates the parameters by maximizing the probability density of the training dataset assigned by $\rho_{{𝐱}^{\prime },{𝐲}^{\prime }}$

The probabilities of $\rho_{y}$ after the inference procedure are given by the following expression:

$$p_{i}^{\prime \prime }=\sum_{\ell =1}^{m}\frac{p_{\ell }p_{i}^{\prime }{\left(k_{𝕏}\left(x^{\left(\ell \right)},x^{\prime (i)}\right)\right)}^{2}}{\sum_{j=1}^{m^{\prime }}p_{j}^{\prime }{\left(k_{𝕏}\left(x^{\left(\ell \right)},x^{\prime (j)}\right)\right)}^{2}},\text{ for }i=1\ldots m^{\prime }$$ (8)

### WiSDoM

WiSDoM is a probabilistic deep learning framework, based on KDM, for automated grading of prostate whole-slide images. It operates in a fully and weakly supervised manner, placing a strong emphasis on interpretability and explainability.

The application of KDM in medical imaging has already been proven effective in domains such as diabetic retinopathy analysis and prostate cancer tissue grading [6]. Its success stems from its unique ability to integrate the robust feature representation of deep convolutional neural networks with a differentiable probabilistic regression model. This integration enables KDM to offer a representation of label probability distributions within an ordinal regression framework. Such a framework is particularly adept at modeling cancer progression as a continuum. A key strength of KDM lies in its ability to predict posterior probability distributions, which allows for the precise quantification of the uncertainty in its predictions.

#### WiSDoM pipeline.

**Fig 1:**
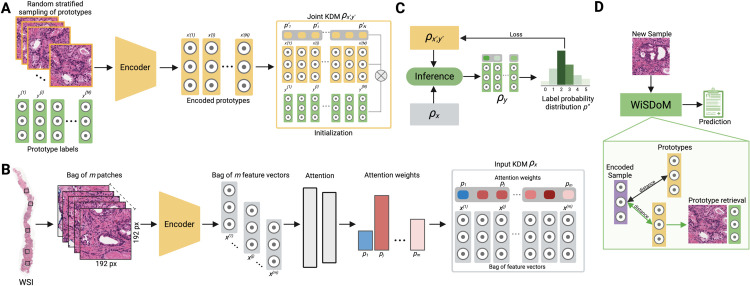
WiSDoM architecture. A. Initialization: The process begins with an initialization step where pairs of samples and labels are encoded to set the parameters of the joint KDM. B. Encoding: For the fully-supervised patch Gleason classifier, individual patches are fed directly, bypassing attention weighting since each patch has a weight of 1. In the weakly-supervised WiSDoM, patch bags are extracted from the WSI, encoded into a feature space by a CNN, and processed through an attention network that aggregates local and global information. These feature vectors and attention weights are represented as a density matrix, modeling the information as an input probability distribution. C. Inference/Training: From the joint KDM of weighted prototypes and their labels, an output distribution of labels is derived, providing a whole slide-level label posterior distribution with an expected value and variance. During training, all parameters in the encoder, attention network, and joint KDM are updated via gradient descent. D. Prototyping: When a new sample is input into WiSDoM, it is first encoded into latent space by the encoder. This encoded feature vector is then compared with all prototypes learned in the joint KDM using Euclidean distance. The closest prototype is used to retrieve the corresponding example and label from the initialization set.

Fig 1 illustrates the WiSDoM general pipeline, applicable to both fully-supervised and weakly-supervised settings. The process begins with initialization, where sample-label pairs are encoded to set the parameters of the joint KDM. This initialization uses encoded patch-label pairs from the training dataset to form the initial KDM $\rho_{{𝐱}^{\prime },{𝐲}^{\prime }}$.

WiSDoM employs a deep neural network as a feature extractor, transforming input patches into 128-dimensional feature vector representations $x\in {\mathbb{R}}^{128}$. In the fully-supervised setting, such as Gleason pattern grading, individual patches are fed directly into the model. The patch feature vectors are represented as a KDM $\rho_{𝐱}$ with *m* = 1 components and $p^{\prime }=1$.

For weakly-supervised tasks, WiSDoM incorporates an attention mechanism. Patch bags extracted from the WSI are processed through an attention network that aggregates local and global information, assigning weights to different patches. The feature vectors, along with attention weights in the weakly-supervised case, are represented as a density matrix, modeling the information as an input probability distribution. This forms the joint KDM of weighted prototypes and their labels. From this joint KDM, WiSDoM derives an output distribution of labels, providing a whole slide-level label posterior distribution with an expected value and variance.

The joint KDM $\rho_{{𝐱}^{\prime },{𝐲}^{\prime }}$ is initialized by an arbitrary set of encoded patch-label pairs $C_{x^{\prime }y^{\prime }}$ from the training dataset *D*. During training, all parameters in the encoder, attention network (for weakly-supervised tasks), and joint KDM are updated via gradient descent.

WiSDoM predicts a probability distribution over ordinal labels, providing expected value and variance for each prediction. The learned internal parameters of the joint KDM allow prototypes to be obtained as examples of the internal learned representations.

The inference procedure involves using the KDM $\rho_{{𝐱}^{\prime },{𝐲}^{\prime }}$ and input KDM $\rho_{𝐱}$, and performing an inference operation (see Eq (8)). The resulting KDM $\rho_{y}$ contains a discrete probability distribution of output labels $p^{\prime \prime }=(p_{1}^{\prime \prime },p_{2}^{\prime \prime },\ldots ,p_{n}^{\prime \prime })$, where each $p_{i}^{\prime \prime }$, represents the probability associated with the *i*-th label. When performing a classification task, we select the most probable label from the distribution. When performing ordinal regression, we slightly modify the inference procedure: First, we convert categorical labels to continuous labels in the range [0,1], the conversion operation from a categorical label to an ordinal label is simply achieved by normalizing the categorical label of each sample by the total number of possible labels as follows (Eq (9)).

$$y_{\text{ordinal}}=\frac{y_{\text{categorical}}}{N_{\text{labels}}}$$ (9)

From the probability distribution obtained from $\rho_{𝐲}$ we can calculate the expected value and variance.

Given a density matrix $\rho_{𝐲}$, represented by a vector $p^{\prime \prime }=(p_{1}^{\prime \prime },p_{2}^{\prime \prime },\ldots ,p_{n}^{\prime \prime })$, where each $p_{i}^{\prime \prime }$ represents the probability associated with the *i*-th label, the expected value and variance are computed as follows:

$$E[\hat{y}{]}_{i}=\sum_{j=1}^{m}p_{ij}^{\prime \prime }\cdot y_{j}^{\prime }$$ (10)

Where ${y_{j}^{\prime }}_{j=1\ldots m}$ are the values associated with each label.

The variance is calculated as the expected value of the squares minus the square of the expected value. This is given by:

$$Var[\hat{y}{]}_{i}=E[{\hat{y}}^{2}{]}_{i}-(E[\hat{y}{]}_{i}{)}^{2}$$ (11)

where $E[{\hat{y}}^{2}{]}_{i}$ is calculated similarly to $E[\hat{y}]$ but using the square of the values ($y_{j}^{\prime 2}$):

$$E[{\hat{y}}^{2}{]}_{i}=\sum_{j=1}^{m}p_{ij}^{\prime \prime }\cdot {y_{j}^{\prime }}^{2}$$ (12)

The expected value and variance are then output for each input patch. Algorithms 1 and 2 summarize the training and prediction procedure of fully-supervised WiSDoM.

**Algorithm 1 Fully-supervised WiSDoM training algorithm.**

**Input:**

$D=\{(x_{i},y_{i}){\}}_{i=1\ldots N}$: Training dataset.

*m*: number of components (encoded set of training patches) of KDM $\rho_{{𝐱}^{\prime },{𝐲}^{\prime }}$

*Z*: Deep learning backbone

1. KDM $\rho_{{𝐱}^{\prime },{𝐲}^{\prime }}$ is initialized with a sample of size *m* from dataset *D*

2. for each $(x_{i},y_{i})\in D$: $\rho_{y}=(\{y_{i}^{\prime }{\}}_{i=1\ldots m},(p_{i}^{\prime \prime }{)}_{i=1\ldots m},K_{Y})=\text{predict}(x_{i},\rho_{{𝐱}^{\prime },{𝐲}^{\prime }},Z)$ (see Algorithm 2)

3. If task = classification: i. $\hat{y}=y_{\arg\max(p^{\prime \prime })}^{\prime }$

ii. Minimize $L=-\sum_{i=1}^{N}y_{i}\log({\hat{y}}_{i})$

4. If task = ordinal regression:

i. Calculate $E[\hat{y}{]}_{i}=\sum_{j=1}^{m}p_{ij}^{\prime \prime }\cdot y_{j}^{\prime }$ and $Var[\hat{y}{]}_{i}=E[{\hat{y}}^{2}{]}_{i}-(E[\hat{y}{]}_{i}){\;}^{2}$, where $E[{\hat{y}}^{2}{]}_{i}=\sum_{j=1}^{m}p_{ij}^{\prime \prime }\cdot y_{j}^{{\;}^{\prime }2}$

ii. Minimize $L=\frac{1}{N}\sum_{i=1}^{N}(E[\hat{y}{]}_{i}-y_{i}){\;}^{2}+\alpha \cdot Var[\hat{y}{]}_{i}$, where *α* is a penalization parameter for variance.

5. Update all backbone weights **w**, and KDM $\rho_{{𝐱}^{\prime },{𝐲}^{\prime }}$ parameters using gradient descent.

6. Return $\left(Z,\rho_{{𝐱}^{\prime },{𝐲}^{\prime }}\right)$

**Algorithm 2 Fully-supervised WiSDoM prediction procedure.**

**Input:**

$\rho_{{𝐱}^{\prime },{𝐲}^{\prime }}=\{(x_{i}^{\prime },y_{i}^{\prime }){\}}_{i=1\ldots m},(p_{i}{)}_{i=1\ldots m},k_{𝕏}⊗k_{𝕐}$: joint KDM

*x*: input patch

*Z*: Deep Learning backbone

1. Encode patch *x* using *Z*: z=Z(x)

2. Create $\rho_{x}=(\{z\},(1),k_{𝕏})$

3. Calculate probabilities $p^{\prime \prime }$ for output KDM $\rho_{y}$ using $\rho_{x}$ and $\rho_{{𝐱}^{\prime },{𝐲}^{\prime }}$ with Eq (8)

4. $\rho_{y}=(\{y_{i}^{\prime }{\}}_{i=1\ldots m},(p_{i}^{\prime \prime }{)}_{i=1\ldots m},K_{Y})$

5. Return $\rho_{y}$

#### Weakly-supervised tasks.

Our prior approach relied on tissue annotations to select relevant patches and to gather Gleason pattern labels for patches across an entire slide. This strategy facilitated an interpretable method for quantifying the extent of each Gleason pattern on the whole slide, similar to how a pathologist would conduct their diagnosis, all centered around WiSDoM classifying patches into specific Gleason patterns.

However, given the high cost and relative unavailability of tissue annotation masks in real-world clinical scenarios, we aim to eliminate the necessity for tissue annotations during training. In this section, we propose a novel method that requires only a whole-slide diagnosis, typically an ISUP grade group for prostate biopsies, which can be easily obtained from pathology reports.

Competitive performance can be achieved in line with current state-of-the-art methodologies for whole-slide grading, which only needs a weak label for training while being constrained by providing interpretability.

We extend WiSDoM probabilistic deep learning framework for weakly supervised, interpretable ordinal regression and classification. It operates on the principle that each WSI in the training set is an individual data point with an established slide-level diagnosis yet lacks specific pixel or region-level annotations. The framework adopts a similar approach to MIL, viewing each WSI as a collection of numerous smaller segments or patches (see Fig 1).

Traditionally, MIL focuses on binary classification, discerning positive from negative classes under the assumption that the presence of one positive patch classifies the entire slide as positive. This approach typically employs a max-pooling aggregation function, choosing the patch with the highest probability of the positive class for slide-level classification. However, this method is unsuitable for multiclass or binary classifications without explicit positive/negative annotations.

WiSDoM differentiates itself by not using the standard max-pooling or other conventional aggregation functions like average pooling, generalized mean, or log-sum-exp, which are limited in terms of problem-specific adaptability and interpretability. Instead, WiSDoM integrates an attention-guided KDM for aggregating information from patches. This method allows for a more nuanced integration of patch-level data into a unified WSI prediction or representation, offering enhanced interpretability and adaptability for various classification problems.

Following tissue detection and patch extraction, WiSDoM involves encoding the *N* patches constituting a WSI into a feature vector representation $x_{n}\in {\mathbb{R}}^{128}$ utilizing a deep learning backbone.

In our study on ISUP grading, we adopt a novel approach by using a collection of sample instances, known as ‘bags,’ instead of labeling each sample individually. This method is particularly suited to scenarios where patches from a whole slide collectively form a specific ISUP grade group, but individual Gleason patterns at the patch level remain unknown, a common occurrence in real-world settings.

We interpret a specific collection of patches from a WSI as a ‘bag.’ The challenge for the model is to learn to assign accurate labels to each patch within these bags and then synthesize this information to make a comprehensive prediction at the whole-slide level. This approach inherently involves uncertainties, especially regarding the individual characteristics of each patch within a bag. Our objective is to model these uncertainties effectively. This integration allows for a more accurate and reliable prediction process, closely mirroring the complexities encountered in actual pathological assessments.

During the training process, WiSDoM takes bags of training samples $X^{\left(i\right)}={\left(x^{(i)j}\right)}_{j=1\ldots m_{i}}$. The training dataset corresponds to a set of pairs $D={\left(X^{\left(i\right)},y^{\left(i\right)}\right)}_{i=1\ldots \ell }$, where each $y^{\left(i\right)}$ is a vector expressing the label proportions of the *i*-th sample. Each input sample is represented by a KDM $\rho_{x}$ with *m*_*i*_ components. For our specific problem, where the goal is to obtain a whole-slide ISUP grade group from a patch bag, we model a variant of the original implementation of KDM [5]. It receives training sample bags as a set of pairs $D={\left(X^{\left(i\right)},y^{\left(i\right)}\right)}_{i=1\ldots \ell }$, where each $y^{\left(i\right)}$ is the ISUP grade group of the bag, perceived as the whole-slide ’weak’ label.

Furthermore, considering the density matrix representation inherent to the KDM, which ascribes a probability to each possible label, we can model the significance or contribution of each instance within a bag towards the overall bag’s label. To accomplish this, we employ a local-global attention method, as shown in [28]. This method assigns a weight, or a contribution factor, to each instance within the bag. Its application to natural images has proven to be useful, as it not only enhances performance but is also able to pinpoint regions of interest (ROIs), providing an additional layer of interpretability. This contribution of each patch to the bag class can be modeled into the probability *p*_*i*_ of each KDM $\rho_{x}$ component $x^{(i)j}$. By incorporating this additional information, we enhance the weakly-supervised learning process by compelling the model to assign greater importance to certain instances within the bags over others.

This attention module receives patches from a bag and processes it using two multi-layer perceptrons (MLPs), which form the means to extract attention weights from these patch bags. The initial MLP is charged with computing the local context, which essentially encapsulates the local information available in each patch ${𝐱}_{j}$. This is accomplished by passing the input through the first MLP, defined as *MLP*_1_, which yields ${𝐳}_{j}^{\text{local}}$.

$${𝐳}_{j}^{\text{local}}=MLP_{1}({𝐱}_{j})$$ (13)

Subsequently, a global context is obtained by aggregating the local context across all patches.

$$z^{\text{global}}=\frac{1}{k}\sum_{j=1}^{k}{𝐳}_{j}^{\text{local}}$$ (14)

Where *k* is the number of patches in the bag. This step provides understanding of the information present in the input data and forms the basis for the subsequent attention distribution. The local (${𝐳}_{j}^{\text{local}}$) and global ($z^{\text{global}}$) information are then combined, and this representation of both local and global information, are local-global embeddings that are fed to the second MLP *MLP*_2_, yielding another set of weights, **z** which are the importance of each patch in the bag. The raw attention weights, **z**, are then passed through a Softmax operation.

$${𝐳}_{j}^{\text{attn}}=\text{Softmax}(MLP_{2}(({𝐳}_{j}^{\text{local}},{𝐳}_{\text{global}})))$$ (15)

The final attention weights are ${𝐳}_{j}^{\text{attn}}$ for each patch in the bag. This Softmax operation normalizes these weights such that they all lie between 0 and 1 and their total sum equals 1. The application of this operation allows the model to weigh each patch based on both the unique contribution of each patch and the global context, enhancing the model’s performance by considering both individual and collective factors.

The primary differentiation in this approach, in comparison to the previous experiment, resides in the KDM $\rho_{x}$ creation process. Instead of uniformly distributing weights by assigning $\frac{1}{m_{i}}$ to every patch in the bag, where *m*_*i*_ is the total number of patches, this novel approach utilizes the attention mechanism to determine these weights. This inclusion allows for a more informative weight assignment that takes into account both individual patch contributions and their collective influence: we assign $p={𝐳}^{\text{attn}}$ in $\rho_{x}$. Each MLP is configured with 64 neurons and uses a ReLU activation function. This configuration, with the number of neurons being half of the input’s dimension, is chosen based on the feature vector size of 128 neurons, effectively reducing the input dimensionality by half, balancing between model complexity and computational efficiency, ensuring that the model is capable of learning a rich set of features without being prohibitively expensive to train on top of the encoder and KDM $\rho_{{𝐱}^{\prime },{𝐲}^{\prime }}$ parameters. The training is conducted in an end-to-end manner, optimizing the parameters across all components of the model. This includes the patch encoder, the global-local attention mechanism, and the KDM $\rho_{{𝐱}^{\prime },{𝐲}^{\prime }}$.

Additionally, the trained local-global attention layer of our model can be extended to provide qualitative interpretability of the decisions, not only providing a way to visualize the most important patches in the patch bag but effectively showing the most significant patches in the slide for accurately prediction its ISUP grade group.

The core of the slide-level classification task is the inference process using the KDM $\rho_{{𝐱}^{\prime },{𝐲}^{\prime }}$ and input KDM $\rho_{x}$ in the same fashion as the fully-supervised case using Eq (8).

The density matrix $\rho_{y}$ is then translated into a discrete probability distribution over the classes. A vector of probabilities is computed from the components of $\rho_{y}$, where the weights and vectors are denoted by $p^{\prime \prime }$ and $y^{\prime }$, respectively. Both are normalized, $p^{\prime \prime }=\frac{p^{\prime \prime }}{\sum p^{\prime \prime }}$ and $y^{\prime }=\frac{y^{\prime }}{\left\|y^{\prime }\right\|}$, and the probability distribution is obtained as $p^{\prime \prime }=\sum_{j}p_{j}^{\prime \prime }y_{ji}^{\prime 2}$. This probability vector represents the likelihood of the WSI belonging to each class, forming the basis for the slide-level classification or ordinal regression task. For the ordinal regression task, we add a final regression layer that takes the probability distribution of labels $p^{\prime \prime }$ as input. This layer computes the expected value and variance for predictions. Algorithms 3 and 4 show a summary of the training and prediction procedure of WiSDoM in a weakly-supervised setting.

**Algorithm 3 Weakly-supervised WiSDoM training algorithm.**

**Input:**

$D=\{(X^{\left(i\right)},y^{\left(i\right)}){\}}_{i=1\ldots N}$: Training dataset, with $X^{\left(i\right)}=\{X_{j}^{\left(i\right)}{\}}_{j=1..k}$ a WSI with *k* patches

*m*: number of components of KDM $\rho_{{𝐱}^{\prime },{𝐲}^{\prime }}$

*Z*: Deep learning backbone

1. KDM $\rho_{{𝐱}^{\prime },{𝐲}^{\prime }}$ is initialized with a sample of size *m* from dataset *D*

2. for each $(X^{\left(i\right)},y^{\left(i\right)})\in D$: $\rho_{y}=(\{y_{i}^{\prime }{\}}_{i=1\ldots m},(p_{i}^{\prime \prime }{)}_{i=1\ldots m},k_{𝕐})=\text{predict}(X^{\left(i\right)},\rho_{{𝐱}^{\prime },{𝐲}^{\prime }},Z)$ (see Algorithm 4)

3. If task = classification:

i. $\hat{y}=y_{\arg\max(p^{\prime \prime })}^{\prime }$

ii. Minimize $L=-\sum_{i=1}^{N}y_{i}\log({\hat{y}}_{i})$

4. If task = ordinal regression:

i. Calculate $E[\hat{y}{]}_{i}=\sum_{j=1}^{m}p_{ij}^{\prime \prime }\cdot y_{j}^{\prime }$ and $Var[\hat{y}{]}_{i}=E[{\hat{y}}^{2}{]}_{i}-(E[\hat{y}{]}_{i}){\;}^{2}$, where $E[{\hat{y}}^{2}{]}_{i}=\sum_{j=1}^{m}p_{ij}^{\prime \prime }\cdot y_{j}^{{\;}^{\prime }2}$

ii. Minimize $L=\frac{1}{N}\sum_{i=1}^{N}(E[\hat{y}{]}_{i}-y_{i}){\;}^{2}+\alpha \cdot Var[\hat{y}{]}_{i}$, where *α* is a penalization parameter for variance.

5. Update backbone, MLP_1_ and MLP_2_ weights **w**, and KDM $\rho_{{𝐱}^{\prime },{𝐲}^{\prime }}$ parameters using gradient descent.

6. Return $\left(Z,\rho_{{𝐱}^{\prime },{𝐲}^{\prime }}\right)$

**Algorithm 4 Weakly-supervised WiSDoM prediction procedure.**

**Input:**

$X=\{x_{j}{\}}_{j=1..k}$: input WSI with *k* patches

$\rho_{{𝐱}^{\prime },{𝐲}^{\prime }}=\{{\left(x_{i}^{\prime },y_{i}\right)}_{i=1\ldots m},(p_{i}{)}_{i=1\ldots m},k_{𝕏}⊗k_{𝕐}\}$: joint KDM

*Z*: Deep Learning backbone

1. ${𝐳}_{j}^{\text{local}}=MLP_{1}({𝐱}_{j})$

2. $z^{\text{global}}=\frac{1}{k}\sum_{j=1}^{k}{𝐳}_{j}^{\text{local}}$

3. ${𝐳}_{j}^{\text{attn}}=\text{Softmax}(MLP_{2}(({𝐳}_{j}^{\text{local}},{𝐳}_{\text{global}})))$

4. Encode patches using *Z*: $z_{j}=Z(x_{j})$

5. Create $\rho_{x}=(\{z_{j}{\}}_{j=1\ldots k},({𝐳}_{j}^{\text{attn}}{)}_{j=1\ldots k},k_{𝕏})$

6. Calculate probabilities $p^{\prime \prime }$ from output KDM $\rho_{y}$ using $\rho_{x}$ and $\rho_{{𝐱}^{\prime },{𝐲}^{\prime }}$ with Eq (8)

7. $\rho_{y}=(\{y_{i}^{\prime }{\}}_{i=1\ldots m},(p_{i}^{\prime \prime }{)}_{i=1\ldots m},K_{Y})$

8. Return $\rho_{y}$

### Training details

During training, we extract a set of patches from each slide. We use automatic tissue detection to identify areas with tissue content and randomly select patches with more than 90% tissue content. We select 36 patches at 20x magnification, each measuring 192×192 pixels, for each whole-slide image. We use a pretrained ConvNeXT [29] as the deep learning encoder to map patch bags to latent space. The network undergoes a warm-up for 2 epochs by processing patches in a classification task, then we attach this backbone to the KDM with adjusted weights post-warm-up.

#### Prototype initialization and selection.

For KDM initialization, the joint KDM $\rho_{{𝐱}^{\prime },{𝐲}^{\prime }}$ requires *m* = 216 components, with 36 prototypes for each of the 6 ISUP grade groups (0-5). We randomly select 36 samples from each class through stratified sampling from the training dataset.

During initialization, each selected prototype is processed through the pre-warmed ConvNeXT encoder to obtain 128-dimensional feature representations. These encoded representations initialize the component set $C=\{x^{\left(1\right)},\ldots ,x^{\left(m\right)}\}$ of the joint KDM, where $x^{\left(i\right)}\in {\mathbb{R}}^{128}$ represents the encoded prototype. The corresponding labels $\left\{y^{\left(1\right)},\ldots ,y^{\left(m\right)}\right\}$ are one-hot encoded representations for classification tasks or normalized continuous values for ordinal regression tasks. The prototype mixture weights are initialized uniformly as $p_{i}=\frac{1}{m}$ for all components.

During training, the components of $\rho_{{𝐱}^{\prime },{𝐲}^{\prime }}$ undergo refinement through gradient-based optimization. The prototype positions $x^{\left(i\right)}$ in the 128-dimensional latent space evolve to maximize class separability according to the RBF kernel $k_{\theta }$. The mixture weights $p=(p_{1},\ldots ,p_{m})$ are learned simultaneously, with the optimization process determining which prototypes contribute most to the inference operations defined in Eq 8. Post-training analysis shows that the model utilizes a subset of the initialized prototypes. Components with learned weights below *p*_*i*_<0.01 contribute minimally to the final predictions, indicating that the KDM framework selects the most discriminative prototypes for each diagnostic category.

#### Computational complexity analysis.

We select *m* = 216 prototypes to balance representational capacity with computational efficiency. With 36 prototypes per ISUP grade group (6 groups total), this configuration provides diversity within each diagnostic category while maintaining tractable inference complexity. Table 1 shows the computational impact of different prototype configurations.

**Table 1:** Computational analysis of prototype count impact.

*m* (Prototypes)	KDM Parameters	% of Total Model	Kernel Evaluations
108	14,580	0.05%	3,888
216	29,160	0.10%	7,776
432	58,320	0.21%	15,552
648	87,480	0.31%	23,328

Kernel evaluations calculated for *n* = 36 patches per whole-slide image. Total model parameters: 27,989,130. KDM parameters include prototype positions (m×128) and corresponding labels (m×6).

The computational complexity of KDM inference scales as 𝒪(n×m) where *n* is the number of input patches and *m* is the prototype count, following Eq (8). The KDM parameters (m×128 for prototype positions plus m×6 for label encodings) constitute 0.10% of the total model parameters with *m* = 216 prototypes, while the encoder backbone represents 99.90% of model complexity. Prototype scaling has minimal impact on model size but linear impact on inference operations through the kernel evaluations $k_{\theta }(x^{\left(\ell \right)},x^{\left(i\right)})$ required for the inference procedure in Eq (8).

#### Optimization and training procedure.

After initializing the KDM $\rho_{{𝐱}^{\prime },{𝐲}^{\prime }}$, the deep learning backbone, attention module, and KDM parameters $\left(C,p,k_{\theta }\right)$ are trained end-to-end. We use the Adam [30] optimizer with a learning rate of $1\;\times \;10^{-4}$, $\beta_{1}=0.9$, and $\beta_{2}=0.999$. A gradual warm-up scheduler with a factor of 10 is applied for 1 epoch, followed by cosine annealing for the remaining epochs. The mini-batch size is set to 4 bags. For the loss function, we use categorical cross-entropy for the classification task. The model is trained for 50 epochs with an early-stopping callback to prevent overfitting, stopping training after 5 epochs without validation loss improvement.

For the ordinal regression task, the warm-up and KDM initialization follow the same procedure. However, we use real-valued labels normalized to the range [0,1] during training instead of one-hot encoded labels, following Eq (9). The loss function is modified to Mean Squared Error with an additional penalization *α* for high variance predictions as shown in Algorithm 3. The same Adam optimizer settings are employed, along with a gradual warm-up scheduler and a mini-batch size of 4 bags.

## Results

### Dataset description

We evaluated WiSDoM on two tasks: (1) fully supervised Gleason pattern classification at patch level and (2) weakly supervised ISUP grade group prediction at the whole slide level. For both tasks, we used the Prostate Cancer Grade Assessment (PANDA) dataset [31], which contains 10,616 deidentified images of the entire slide from two institutions:

- Radboud University Medical Center (D1): Scanned at 20× magnification (0.24 *μ*m/pixel) using 3DHistech Pannoramic Flash II 250
- Karolinska Institutet (D2): Scanned at 20× magnification (0.45-0.50 *μ*m/pixel) using Hamamatsu C9600-12 and Aperio ScanScope AT2

All digital images of archived samples were de-identified before being made publicly available for research and publication following the publication of the main challenge article [31]. The data were accessed through the Kaggle platform in June 2022, which hosted the deidentified whole-slide images. The original PANDA study received approval from multiple institutional review boards: Radboud University Medical Center (IRB 2016-2275), Stockholm regional ethics committee (permits 2012/572-31/1, 2012/438-31/3 and 2018/845-32), and Advarra in Columbia, MD (Pro00038251). While participants in the Swedish dataset provided informed consent, the requirement was waived for other datasets due to their use of de-identified prostate specimens in a retrospective context.

D1 provides pixel-level annotations for Gleason patterns (3, 4, 5), stroma, and healthy tissue, annotated by consensus of pathology-trained medical students. D2 contains binary annotations (cancerous/non-cancerous) from an expert pathologist. After quality control, we excluded 1,956 slides from processing.

For patch-level classification, we extracted patches exclusively from D1 because of its detailed annotations. Patches were assigned to a class if they contained >25% of that tissue type. For whole-slide classification, we used data from both D1 and D2. We maintained consistent train-validation-test splits at the slide level to prevent data leakage. Dataset composition details are provided in Supplementary Tables 1-3.

**Table 2:** Quantification of average Gleason pattern extension in whole-slides grouped by ISUP grade group. True extension is measured from each healthy epithelium, stroma, and Gleason pattern available tissue annotations. Predicted extension is calculated by performing inference at a patch level with a high overlapping ratio of patches. The difference in extend is then quantified using MAE per tissue pattern and then averaged across all slides in each ISUP grade group.

ISUP Grade Group	Gleason Pattern	True extension %	Predicted %	MAE
**GG 0**	**Stroma**	**78.0**	**76.5**	**0.0059**
	Healthy Epithelium	22.0	22.8	
	Gleason 3	0.0	0.04	
	Gleason 4	0.0	0.02	
	Gleason 5	0.0	0.01	
**GG 1**	**Stroma**	**64.4**	**62.3**	**0.0131**
	Healthy Epithelium	10.7	13.4	
	Gleason 3	24.9	23.7	
	Gleason 4	0.0	0.05	
	Gleason 5	0.0	0.0	
**GG 2**	**Stroma**	**55.6**	**51.9**	**0.0171**
	Healthy Epithelium	5.4	7.3	
	Gleason 3	27.1	29.1	
	Gleason 4	11.9	11.3	
	Gleason 5	0.0	0.04	
**GG3**	**Stroma**	**57.2**	**54.0**	**0.0165**
	Healthy Epithelium	5.7	7.4	
	Gleason 3	9.7	11.2	
	Gleason 4	27.4	26.5	
	Gleason 5	0.0	0.09	
**GG 4**	**Stroma**	**62.8**	**60.6**	**0.0117**
	Healthy Epithelium	5.1	6.1	
	Gleason 3	2.6	3.5	
	Gleason 4	25.9	25.2	
	Gleason 5	3.5	4.7	
**GG 5**	**Stroma**	**62.8**	**59.3**	**0.0174**
	Healthy Epithelium	3.0	3.4	
	Gleason 3	0.0	0.08	
	Gleason 4	21.6	20.7	
	Gleason 5	12.6	15.8	
**Overall**				0.0136

**Table 3:** Performance Metrics for Whole-Slide and Patch-Level Grading, and Comparison with PANDA Consortium Teams

(a) Whole-Slide Grading (ISUP)			
**WiSDoM**	** κ **	**Accuracy**	**MAE**
Whole-Slide Classification	0.898	0.663	-
Ordinal Regression (Whole-Slide)	0.900	0.660	0.173
Ordinal Regression with Variance Threshold ($\sigma^{2}<0.05$)	0.930	0.73	0.073
**(b) Patch-Level Grading (Gleason Pattern)**			
**WiSDoM**	** κ **	**Accuracy**	**MAE**
Five-Class Classification	0.896	0.901	-
Ordinal Regression	0.906	0.890	0.13
Ordinal Regression with Variance Threshold ($\sigma^{2}<0.05$)	0.924	0.910	0.13
**(c) Comparison with PANDA Consortium Teams**			
**Team Name**	** κ **		
Dmitry A. Grechka	0.8861		
KovaLOVE v2	0.8889		
ctrasd123	0.8948		
Manuel Campos	0.8980		
Kiminya	0.9007		
PND	0.9108		
BarelyBears	0.9118		
ChienYiChi	0.9086		
rähmä.ai	0.9096		
iafoss	0.9179		
NS Pathology	0.9180		
Save The Prostate	0.9209		
**Ours**	**0.9300**		

Note: Our score was calculated using the test partition from the open development dataset as described in the dataset description section.

### Fully-supervised patch Gleason grading

In five-class Gleason pattern classification (stroma, benign epithelium, Gleason 3, 4, and 5), WiSDoM achieved κ=0.896 and accuracy = 0.901. For ordinal regression, the model achieved κ=0.906, accuracy = 0.890, and MAE = 0.13.

When filtering predictions to those with low variance ($\sigma^{2}<0.05$), performance improved to κ=0.924 and accuracy = 0.910, maintaining MAE = 0.13 (Table 3b). Supplementary Figure 1a shows the relationship between prediction errors and variance in the test set.

WiSDoM estimated the percentage of each tissue type (Stroma, Healthy Epithelium, Gleason 3, 4, and 5) within WSIs per Grade Group. The overall MAE was 0.0136 across tissue types. MAE values ranged from 0.0059 in Grade Group 0 (benign) to 0.0174 in Grade Group 5 (highest grade). Complete area estimation comparisons for all Grade Groups are provided in Table 2.

### Weakly-supervised whole-slide grading

For weakly-supervised whole-slide ISUP grade prediction, WiSDoM achieved κ=0.898 and accuracy = 0.663 in classification. The ordinal regression yielded κ=0.900, accuracy = 0.660, and MAE = 0.173. Filtering predictions by variance threshold ($\sigma^{2}<0.05$) resulted in κ=0.930, accuracy = 0.73, and MAE = 0.073 (Table 3a).

Table 3c compares WiSDoM with PANDA Challenge teams. The highest scoring submission, “Save The Prostate," achieved κ=0.9209. WiSDoM achieved κ=0.9300. The complete performance metrics for all teams are provided in Table 3c.

### Model interpretability and uncertainty analysis

WiSDoM produces three types of interpretable outputs: attention heatmaps, prototype examples, and uncertainty measurements.

**Fig 2:**
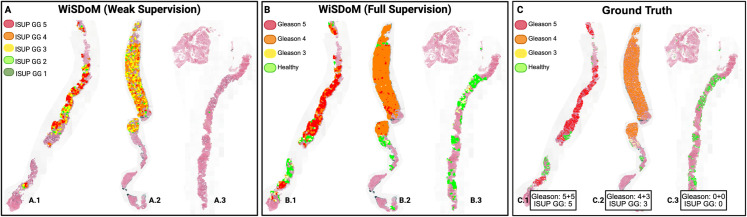
Comparison of visually interpretable heatmaps across fully and weakly supervision levels of WiSDoM. The figure presents heatmaps from three different supervision approaches across multiple whole slide images (WSIs). A) WiSDoM with weak supervision shows predictions for ISUP grade groups across three slides: A.1 (ISUP GG 5), A.2 (ISUP GG 4), and A.3 (ISUP GG 2). B) WiSDoM with full supervision displays Gleason pattern predictions for the same slides: B.1 (Gleason 5), B.2 (Gleason 4), and B.3 (Gleason 3 and healthy tissue). C) Ground truth annotations from pathologists are shown for comparison: C.1 (Gleason 5+5, ISUP GG 5), C.2 (Gleason 4+3, ISUP GG 3), and C.3 (Gleason 0+0, ISUP GG 0). The weakly supervised model (A) predicts ISUP grade groups without requiring detailed patch-level annotations during training, while the fully supervised model (B) provides Gleason pattern predictions trained on patch-level labeled data.

**Attention Heatmaps:** In fully-supervised classification, the heatmaps display Gleason pattern distributions across whole slides with region-level detail. In weakly-supervised classification, the heatmaps indicate regions contributing to ISUP grade predictions without specifying individual Gleason patterns. Fig 2 shows heatmaps from both supervision levels across multiple whole slide images.

**Prototype analysis and clinical validation:** The kernel density framework learns 216 prototypes distributed across the six ISUP grade groups, with 36 prototypes per class randomly selected during initialization and subsequently refined through gradient-based optimization in the 128-dimensional latent space. Post-training analysis reveals that learned prototypes capture diagnostically relevant tissue patterns used in clinical practice.

To validate clinical relevance, we conducted blind assessment by three resident pathologists and one expert urological pathologist. The evaluation included 36 WiSDoM-generated prototypes presented without labels in the context of corresponding whole slide images. Pathologist assessment of prototype labels achieved substantial agreement with model classifications (κ=0.88), reaching inter-pathologist consistency levels and indicating that learned representations correspond to recognized diagnostic patterns.

**Fig 3:**
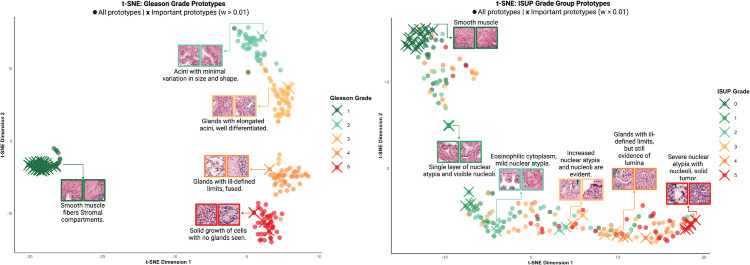
Learned prototypes feature space. t-distributed Stochastic Neighbor Embedding (t-SNE) plot of the learned prototypes inside WiSDoM for different supervision scenarios. In the fully supervised model (left), the prototypes perfectly discriminate the latent space in Gleason grades. Notably, Gleason grade 0 samples are well separated from the other grades and show greater variability, reflecting the higher histological heterogeneity in benign tissue. The t-SNE projection of Gleason grades 1 to 4 suggests a continuum progression of severity (top-down), with these grades showing less variability and thus requiring fewer prototypes to represent them. In the weakly-supervised model (right), not all prototypes are discriminant of the latent space. This reduced separability is expected, as the weak supervision is based on ISUP grades of whole slides, which inherently contain mixtures of local Gleason patterns in varying proportions. Despite this challenge, prototypes with weights or importance over 0.01 (marked with x) can still efficiently differentiate ISUP grades in the latent space, maintaining the pattern of higher variability in lower grades and more defined, less variable representations in higher grades. This demonstrates the model’s ability to learn meaningful representations even with less granular supervision.

**Fig 4:**
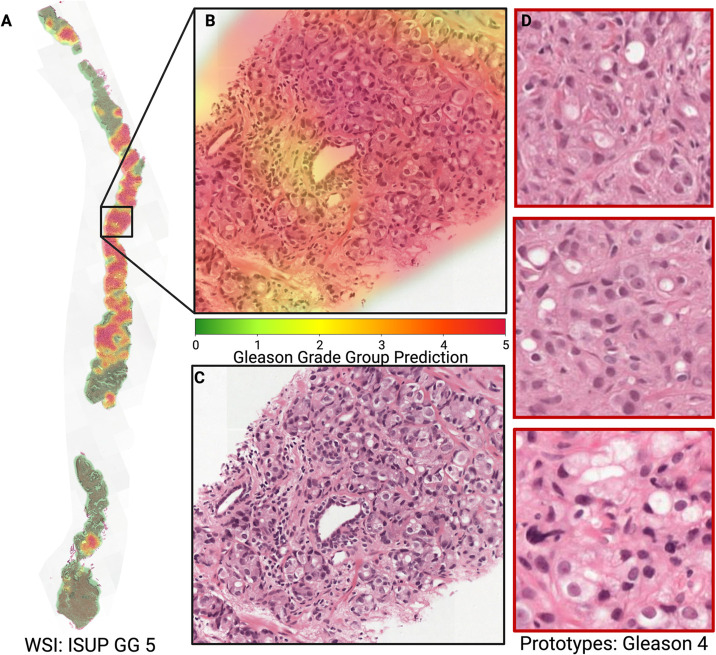
Prototype-based model explainability. The figure shows a region of interest highlighted by the model with a heatmap where colors represent different ISUP grade groups: green for lower grades, progressing through yellow and orange to red for higher grades. Example prototypes are sampled from WiSDoM’s learned representation, each labeled with its corresponding Gleason grade. The highlighted region and relevant prototypes provide visual insight into the model’s decision-making process.

Fig 3 visualizes the learned prototype space through t-SNE embedding, showing the distribution of prototypes across Gleason and ISUP grades and their correspondence to training patches. For two clinically important prototypes, pathologist descriptions highlight the model’s ability to capture both canonical patterns and morphological variants that experts recognize but are difficult to systematize. The spatial organization in the embedding space reflects the ordinal relationship between grades, with adjacent Gleason or ISUP grade groups clustering together while maintaining distinct diagnostic boundaries. Fig 4 illustrates the model’s prototype-based explainability using heatmaps and example prototypes labeled by Gleason grade.

**Fig 5:**
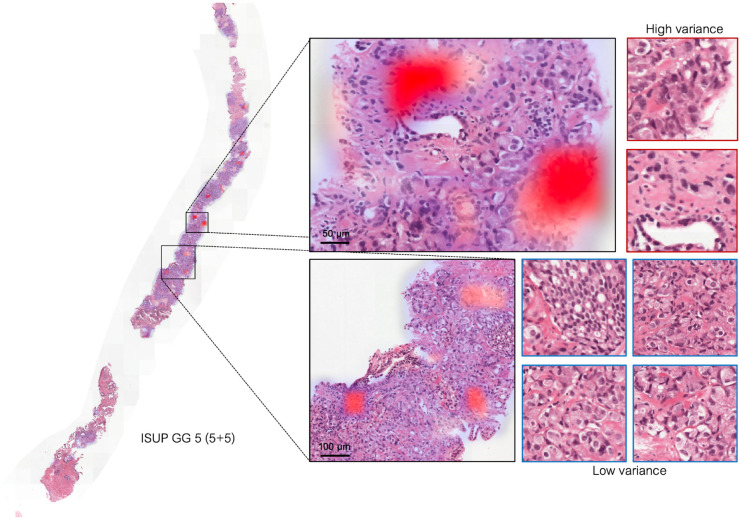
Model uncertainty visualization. A representative slide is shown with regions of high variance highlighted in red. The whole-slide heatmap was generated by obtaining the variance values for the prediction over patches tiled at 80% overlap, with zoomed-in regions on the right. Patches with a red border indicate regions where the model’s uncertainty of the prediction was high, while blue borders indicate high confidence in the prediction.

**Uncertainty Quantification:** WiSDoM produces uncertainty maps by computing prediction variance over overlapping patches across whole slides. Fig 5 shows these maps, where high variance regions (red) indicate areas of prediction inconsistency. The model produces posterior probability distributions over possible classes, variance measurements for each predicted region, and spatial uncertainty maps highlighting regions of low confidence.

## Discussion

Current deep learning methods in computational pathology face a fundamental trade-off: fully-supervised approaches require extensive annotations while weakly-supervised methods sacrifice interpretability. This limitation particularly affects clinical adoption where both performance and transparency are essential. WiSDoM addresses this challenge by demonstrating that kernel density matrices can unify supervision modes while maintaining interpretability and uncertainty quantification, achieving κ=0.930 on whole-slide ISUP grading.

WiSDoM’s performance exceeds the top PANDA Challenge submission (κ=0.921) despite using a single model compared to the ensemble methods with EfficientNet [32] architectures, test-time augmentation, and pseudo-labeling employed by leading teams. More remarkably, when compared to foundation models like UNI [33] that achieve κ=0.946 after pretraining on millions of whole-slide images, WiSDoM demonstrates competitive performance while requiring substantially fewer training examples and providing built-in interpretability mechanisms that foundation models lack. This data efficiency represents an advantage for institutions with limited computational resources or smaller datasets.

The clinical relevance of our approach becomes evident through Cohen’s Kappa comparison with pathologist agreement levels. For patch-level Gleason grading, our κ=0.924 reaches inter-pathologist consistency on the PANDA dataset, while ordinal regression outperforms classification (κ=0.906 vs κ=0.896), reflecting the clinical understanding that adjacent grades represent similar disease severity. This ordinal formulation proves valuable where prediction error severity matters (i.e., misclassifying ISUP 2 versus 3 carries different implications than ISUP 1 versus 5 errors).

WiSDoM’s approach differs from existing weakly-supervised pathology methods. While CLAM [4] pioneered attention-based multiple instance learning, it lacks the probabilistic uncertainty quantification that WiSDoM provides through kernel density matrices. TransMIL [34] and other transformer-based approaches capture long-range dependencies but require post-hoc interpretation methods, whereas WiSDoM provides uncertainty estimates directly through probability distributions without additional calibration. Recent work has shown that supervised pretraining significantly outperforms random initialization across pathology tasks [35], validating our approach of leveraging learned representations, though WiSDoM addresses the complementary challenge of learning meaningful patch-level representations through ordinal regression rather than simple feature aggregation.

The attention mechanism in WiSDoM serves dual purposes compared to other MIL models. Rather than simply aggregating patch features for slide-level classification, our attention weights correspond directly to diagnostic confidence through the probabilistic foundation of kernel density matrices. This connection between attention and uncertainty provides more reliable explanations than gradient-based attribution methods, which exhibit instability across different runs.

Validation from pathologist assessment of learned prototypes, achieved substantial agreement with model classifications (κ=0.88) at inter-pathologist consistency levels. This demonstrates that learned representations correspond to recognized diagnostic patterns rather than arbitrary feature embeddings. Analysis reveals that prototypes capture both canonical histological patterns and morphological variants that experts recognize but are difficult to systematize in textbooks. The spatial organization in learned embedding space reflects the ordinal relationship between grades, with adjacent Gleason groups clustering together while maintaining diagnostic boundaries.

An intriguing takeaway we found was that models trained with slide-level labels learn to identify similar regions as those trained with detailed annotations. Attention heatmaps from fully-supervised and weakly-supervised models show consistent focus on diagnostically relevant areas despite the weakly-supervised model receiving only slide-level labels during training. This convergence validates a key assumption in weakly-supervised learning, that global labels contain sufficient signal for local pattern discovery. The attention mechanism learns to weight patches based on diagnostic relevance without explicit patch-level guidance, maintaining spatial coherence across tissue regions.

Error analysis reveals model difficulties that align with recognized diagnostic challenges. Primary confusion occurs between morphologically similar adjacent grade groups where architectural features show gradual transitions. For the clinically critical ISUP Grade Group 2 versus 3 distinction (Gleason 3+4 versus 4+3), the model correctly classified 42% of Grade Group 2 cases and 37% of Grade Group 3 cases, with bidirectional confusion reflecting the inherent difficulty of quantifying Gleason pattern proportions. This mirrors documented challenges in pathologist interpretation, particularly with borderline pattern proportions, suggesting the model learns genuine tissue relationships rather than arbitrary classifications. Unlike foundation models requiring extensive pretraining data, WiSDoM achieves competitive performance with smaller datasets through ordinal regression that leverages inherent structure in diagnostic grades. The probabilistic regression approach naturally handles ordinal relationships, unlike classification methods treating grades as independent categories.

Computational analysis reveals interesting trade-offs. Prototype count impacts inference complexity through kernel evaluations, with our choice of prototypes balancing representational capacity and efficiency. The computational bottleneck lies primarily in attention mechanisms and backbone feature extraction rather than kernel density operations themselves, making the method feasible where interpretability requirements justify computational overhead.

However, important limitations warrant consideration. The assumption that local patterns aggregate meaningfully to global classification proves well-suited for prostate cancer grading, where diagnostic decisions depend on pattern composition and distribution. Yet this may not generalize to applications requiring detection of rare cellular events or single-cell resolution features. The kernel density framework provides flexibility for different aggregation strategies, but validation across diverse pathology applications remains necessary.

Clinical translation faces implementation challenges beyond technical performance. Model validation requires prospective studies, regulatory approval, and quality assurance protocols. The interpretability features require pathologist training for effective clinical utilization. Integration with existing laboratory systems represents practical deployment considerations, though the unified supervision framework provides regulatory advantages by enabling validation with both detailed annotations for algorithm verification and routine diagnoses for clinical validation.

Future research directions include adaptive prototype selection for optimized efficiency, extension to multi-task learning for simultaneous prediction of multiple pathological features while preserving interpretability, and integration with foundation models to combine their generalization capabilities with WiSDoM’s transparency. The demonstrated ability to maintain interpretability across supervision modes while achieving competitive performance suggests that kernel density matrices offer a promising foundation for clinical AI systems requiring both accuracy and explainability.

### Code availability

Patches from WSI were generated locally using HistoPrep [36]. WiSDoM training was conducted on NVIDIA A100 GPUs on Google Colab Pro. Our pipeline, implemented in Python (3.11), utilizes OpenSlide, Pillow, and TensorFlow v2.

## Supporting information

S1 File Supporting Tables and Figures. This file contains three tables and two figures. S1 Table provides the PANDA dataset description (percentage), where GG = ISUP Grade Group. S2 Table shows the patch dataset distribution, where G = Gleason grade. S3 Table presents the slide dataset distribution, where GG = ISUP Grade Group. S1 Fig displays the distribution of prediction variance versus absolute error in test set samples: (A) Patch-level fully supervised classification showing variance distribution for Gleason pattern predictions (Healthy, G3, G4, G5), and (B) Slide-level weakly-supervised classification showing variance distribution for ISUP grade predictions (0-5). Absolute error represents the distance between predicted and true classes: 0 for correct predictions, 1 for adjacent class errors, and 2+ for errors spanning multiple classes. The violin plots demonstrate increased variance correlates with higher prediction error in both supervision modes. S2 Fig shows learned prototypes sampled from WiSDoM to enhance model explainability. For each ISUP grade group, the top three patches closest to the learned prototypes are displayed, selected from WSIs of the corresponding ISUP grade. The rightmost column shows the closest prototype for each grade group in the context of its whole slide, demonstrating that WiSDoM’s internal representation effectively captures the morphological patterns inherent in the Gleason grades constituting each grade group.
(DOCX)
