# Supplementary material for: Interpretable weakly-supervised learning through kernel density matrices: A digital pathology use case
Source: PLoS One. 2025 Nov 5;20(11):e0335826. doi: 10.1371/journal.pone.0335826 (PMC12588513; doi:10.1371/journal.pone.0335826)
Supplement: S1 File — This file contains three tables and two figures. S1 Table provides the PANDA dataset description (percentage), where GG = ISUP Grade Group. S2 Table shows the patch dataset distribution, where G = Gleason grade. S3 Table presents the slide dataset distribution, where GG = ISUP Grade Group. S1 Fig displays the distribution of prediction variance versus absolute error in test set samples: (A) Patch-level fully supervised classification showing variance distribution for Gleason pattern predictions (Healthy, G3, G4, G5), and (B) Slide-level weakly-supervised classification showing variance distribution for ISUP grade predictions (0-5). Absolute error represents the distance between predicted and true classes: 0 for correct predictions, 1 for adjacent class errors, and 2+ for errors spanning multiple classes. The violin plots demonstrate increased variance correlates with higher prediction error in both supervision modes. S2 Fig shows learned prototypes sampled from WiSDoM to enhance model explainability. For each ISUP grade group, the top three patches closest to the learned prototypes are displayed, selected from WSIs of the corresponding ISUP grade. The rightmost column shows the closest prototype for each grade group in the context of its whole slide, demonstrating that WiSDoM’s internal representation effectively captures the morphological patterns inherent in the Gleason grades constituting each grade group. (DOCX) [file pone.0335826.s001.docx]

**Supporting Information for article “Interpretable weakly-supervised learning through kernel density matrices: a digital pathology use case”**

**
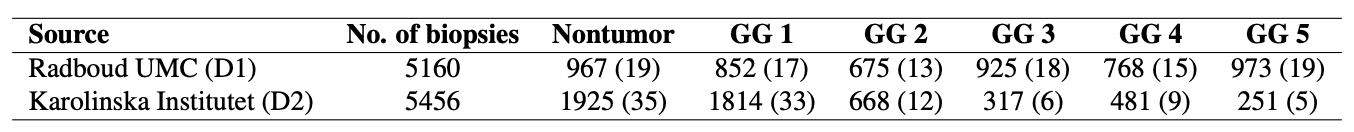
Supplementary Table 1.** PANDA dataset description (Percentage), GG = ISUP Grade Group

**
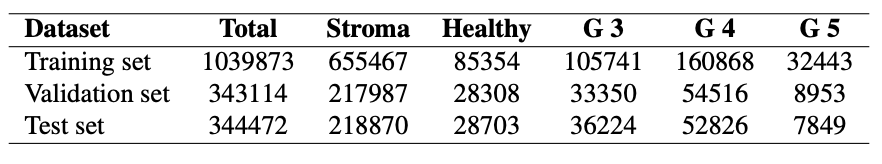
Supplementary Table 2.** Patch dataset distribution, G = Gleason grade

**Supplementary Table 3.** Slide dataset distribution, GG = ISUP Grade Group


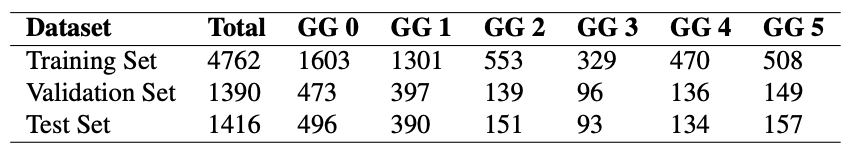


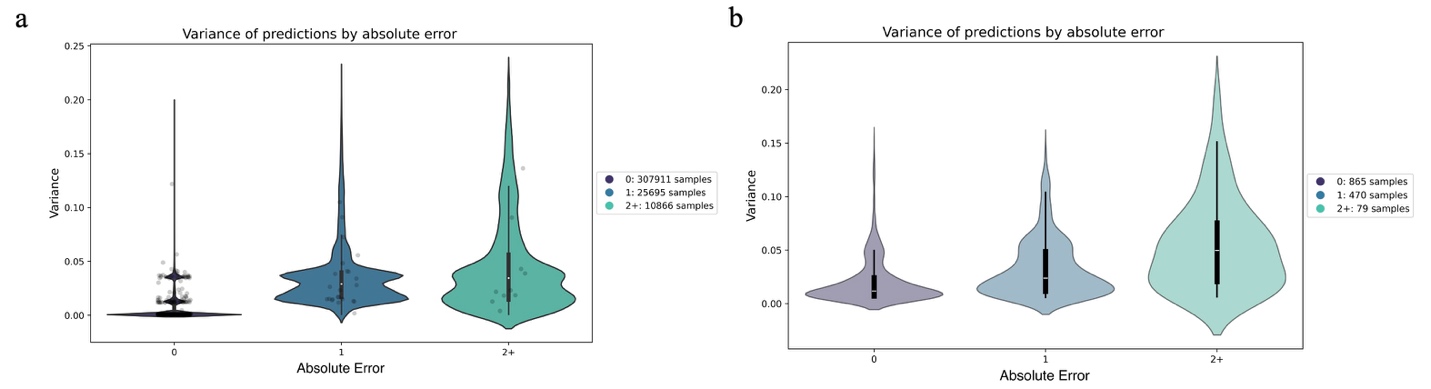


**S1 Fig.** Distribution of prediction variance versus absolute error in test set samples. (A) Patch-level fully supervised classification: variance distribution for Gleason pattern predictions (Healthy, G3, G4, G5). (B) Slide-level weakly-supervised classification: variance distribution for ISUP grade predictions (0-5). Absolute error represents the distance between predicted and true classes: 0 for correct predictions, 1 for adjacent class errors, and 2+ for errors spanning multiple classes. The violin plots demonstrate increased variance correlates with higher prediction error in both supervision modes.


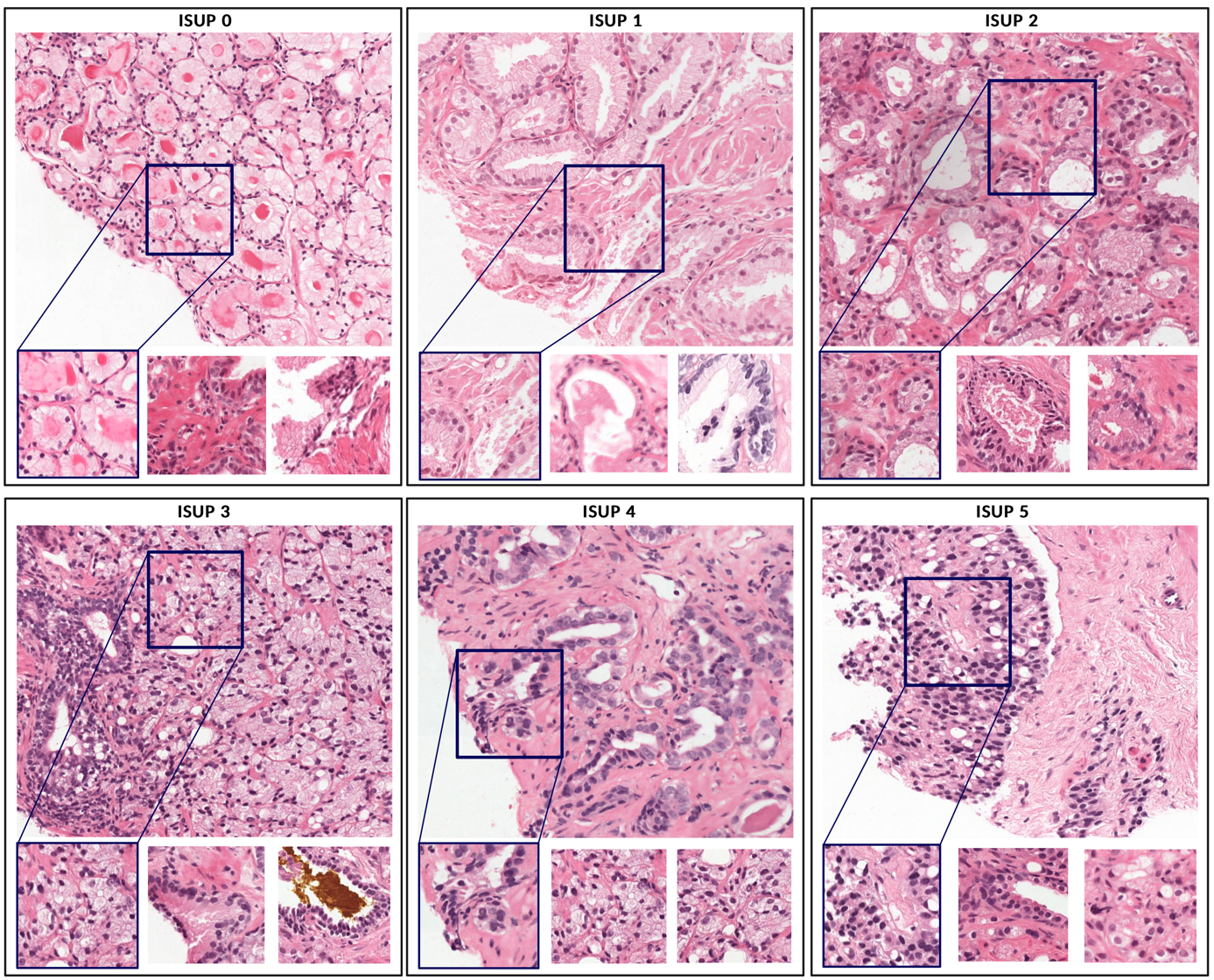


**S2 Fig.** **Learned prototypes.** After training, prototypes are sampled from WiSDoM to enhance model explainability. For each ISUP grade group, we display the top three patches closest to the learned prototypes. These patches are selected from WSIs of the corresponding ISUP grade, ensuring that the prototypes accurately represent the characteristics of each grade group. The rightmost column shows the closest prototype for each grade group in the context of its whole slide. The prototypes demonstrate that WiSDoM's internal representation of ISUP grade groups effectively captures the morphological patterns inherent in the Gleason grades constituting each grade group.
